# Risk Factors and Antibiotic Utilization Patterns in Multidrug‐Resistant Surgical Infections: A Retrospective Study From a Romanian Tertiary‐Care Center

**DOI:** 10.1155/ijm/6286424

**Published:** 2026-06-19

**Authors:** Lucian Ambrosie, Maria Dan, Ovidiu-Dumitru Ilie, Tabita-Daniela Condrea, Mihaela Purice, Mihaela Blaj, Lidia Ionescu, Daniel-Vasile Timofte

**Affiliations:** ^1^ Department of Surgery, “Grigore T. Popa” University of Medicine and Pharmacy, Iasi, Romania, umfiasi.ro; ^2^ Clinical Surgery Department III, “St. Spiridon” Emergency Clinical Hospital, Iasi, Romania; ^3^ Department of Microbiology, “St. Spiridon” Emergency Clinical Hospital, Iasi, Romania; ^4^ Regional Center of Advanced Research for Emerging Diseases, Zoonoses and Food Safety, Department of Public Health, Faculty of Veterinary Medicine, “Ion Ionescu de la Brad” Iasi University of Life Sciences, Iasi, Romania, uaiasi.ro; ^5^ Department of Oral and Maxillofacial Surgery, “St. Spiridon” Emergency Clinical Hospital, Iasi, Romania; ^6^ Department of Anesthesiology and Intensive Care, “Grigore T. Popa” University of Medicine and Pharmacy, Iasi, Romania, umfiasi.ro; ^7^ Anesthesiology and Intensive Care Unit, “St. Spiridon” Emergency Clinical Hospital, Iasi, Romania

**Keywords:** antibiotic prophylaxis, COVID-19, ICU stay, multidrug resistance, risk factors, Romania, surgical infections

## Abstract

**Background:**

Multidrug‐resistant (MDR) infections represent a major challenge in surgical patients, particularly in settings with prolonged hospitalization and intensive care exposure. Identifying risk factors and their impact on mortality is essential for optimizing antimicrobial stewardship and infection control strategies.

**Methods:**

We conducted a retrospective observational study including surgical patients admitted to a Romanian tertiary‐care hospital between 2020 and 2022. Demographic, clinical, microbiological, and antibiotic utilization data were analyzed using multivariate logistic regression and receiver operating characteristic (ROC) curve analysis to identify factors associated with MDR infection and in‐hospital mortality.

**Results:**

Prolonged intensive care unit (ICU) stay and longer hospitalization were the main independent predictors of MDR infection. Antibiotic prophylaxis was also associated with MDR status, likely reflecting increased antimicrobial exposure in high‐risk patients, while a higher comorbidity burden showed an inverse association. MDR infections were strongly associated with increased in‐hospital mortality and remained an independent predictor after adjustment, alongside ICU stay and older age. MDR cases were characterized by more frequent use of broad‐spectrum antibiotics, whereas non‐MDR patients more commonly received narrower‐spectrum regimens. The most frequent MDR pathogens included *Klebsiella pneumoniae*, *Pseudomonas aeruginosa*, and *Acinetobacter baumannii*.

**Conclusions:**

MDR infections in surgical patients are primarily driven by prolonged healthcare exposure, particularly ICU admission and extended hospitalization. These findings highlight the importance of targeted infection control measures and optimized antimicrobial use to reduce mortality and limit the spread of resistance.

## 1. Introduction

Bacterial infections caused by MDR organisms represent an emerging global health crisis. According to a comprehensive analysis by the Antimicrobial Resistance Collaborators, antimicrobial resistance (AMR) was directly responsible for approximately 1.27 million deaths in 2019. MDR bacteria, defined as resistant to at least one agent in three or more antimicrobial classes, continue to spread rapidly worldwide [1]. Their proliferation is driven by well‐documented mechanisms, including chromosomal mutations and horizontal gene transfer [2]. Moreover, the inappropriate use of antibiotics in human healthcare has substantially contributed to the elevation of antibiotic resistance [2].

Global reports from the Antimicrobial Resistance and Use Surveillance System (GLASS) indicate a sustained increase in AMR among bloodstream (BSI) and urinary tract infections (UTI), with nearly one in six participating laboratories reporting critically high rates of carbapenem‐resistant *Enterobacterales* and fluoroquinolone‐resistant *Escherichia coli* [3, 4]. European surveillance data corroborate these findings, showing persistently elevated AMR levels across European Union/European Economic Area (EU/EEA) countries, particularly rising in third‐generation cephalosporin and carbapenem resistance among *Enterobacterales* and high proportions of carbapenem‐resistant *Acinetobacter* spp. [3, 4].

Recent analyses by the European Centre for Disease Prevention and Control (ECDC) further confirm increasing rates of third‐generation cephalosporin‐resistant *E. coli* and carbapenem‐resistant *K. pneumoniae*, alongside an expanding geographic distribution of carbapenem‐resistant *Enterobacterales* in hospital settings [5]. In response, the ECDC has implemented the CRE25 genomic surveillance protocol to enhance the detection and monitoring of carbapenemase‐producing *Enterobacterales* and to support the broader integration of whole‐genome sequencing (WGS) into routine AMR surveillance across Europe [6].

WGS has provided a robust framework for elucidating genomic organization, virulence potential, and host adaptation in bacterial pathogens, frequently revealing compact and highly conserved genomic architectures across diverse hosts and settings [7–9]. Comparative and pangenomic analyses have consistently demonstrated largely clonal population structures, with host specificity and pathogenic variability driven by subtle genetic variations rather than major structural divergence [8, 9]. More broadly, WGS‐based and integrative OMICS approaches have been increasingly applied to identify conserved genes, evolutionary patterns, and pathogenic mechanisms across clinical, veterinary, and environmental pathogens, underscoring their versatility for surveillance and risk assessment [10–12]. In this context, the expanding implementation of WGS in AMR monitoring aligns with recent ECDC initiatives, which leverage genomic sequencing to track the spread of carbapenem‐resistant *Enterobacterales* and other priority pathogens in clinical settings [5, 6].

The burden of AMR is disproportionately higher in low‐ and middle‐income countries (LMICs). Systematic reviews have identified multiple contributing factors that facilitate the development of this resistance, including empirical prescribing practices, limited access to microbiological diagnostics, and unrestricted availability to antibiotics [13]. These circumstances have led to increased treatment failures, prolonged hospital stays, and higher healthcare expenditures [13]. Among hospital‐acquired infections (HAIs), surgical site (SSI) and BSI are particularly susceptible to MDR pathogens, heightening postoperative morbidity [13, 14].

Several key MDR pathogens commonly encountered in clinical settings include *K. pneumoniae*, *A. baumannii*, and *P. aeruginosa*. These pathogens are designated by the World Health Organization (WHO) as critical or high‐priority organisms due to their extensive resistance profiles, frequently mediated by extended‐spectrum *β*‐lactamase (ESBLs) and carbapenemases. Similarly, Gram‐positive pathogens, such as methicillin‐resistant *Staphylococcus aureus* (MRSA) and vancomycin‐resistant *Enterococcus faecium*, also contribute substantially to the hospital‐associated resistance burdens [15]. Infections caused by these MDR pathogens are consistently linked to increased mortality and long‐term disability, underscoring their clinical and public health relevance [1].

National evidence aligns with global AMR trends, with Romanian studies reporting high resistance among Gram‐negative ICU pathogens, including post‐COVID‐19 isolates that often sustain or further amplify pre‐existing resistance patterns [16]. A 4‐year surveillance analysis confirmed escalating resistance across ESKAPE organisms, *E. faecium*, *S. aureus*, *K. pneumoniae*, *A. baumannii*, *P. aeruginosa*, and *Enterobacter* spp., indicating the continued circulation of highly resistant strains in critical care settings [17].

Complementary data from tertiary, transplant, cardiovascular, and secondary‐care hospitals further demonstrate a substantial MDR burden associated with severe clinical outcomes and increased mortality [18–20]. The high prevalence of carbapenemase‐producing *K. pneumoniae* and other last‐resort resistance phenotypes highlights persistent diagnostic limitations and reinforces the need for robust microbiological surveillance and ward‐specific susceptibility data to inform empirical therapy and limit MDR transmission in Romania.

Optimizing empirical antibiotic therapy requires robust local surveillance through tailored antibiograms. Guidelines from major infectious disease societies advocate for stratified antibiograms that incorporate infection site, patient demographics, and hospital ward, rather than relying solely on cumulative susceptibility profiles [21]. Evidence indicates that such approaches, developed collaboratively with clinical microbiologists, are essential for improving antimicrobial stewardship and enhancing patient outcomes [22].

Systematic reviews and meta‐analyses consistently demonstrate that integrated antimicrobial stewardship and infection control interventions effectively reduce inappropriate antimicrobial use and limit the spread of MDR organisms. Evidence from ICU settings, as synthesized by Ntim et al. [23], shows that multifaceted strategies such as audit and feedback, procalcitonin‐guided prescribing, and antimicrobial de‐escalation significantly decrease antibiotic consumption without adversely affecting clinical outcomes. Extending this perspective, the global meta‐analysis by George et al. [24] highlights the substantial burden posed by resistant nosocomial infections and their markedly increased mortality risk, particularly in cases of BSI.

European data further reinforce these findings: analyses by Hassoun‐Kheir et al. [25] and Abdel Hadi et al. [26] document both the excess morbidity and mortality attributable to resistant pathogens and the consistent benefits of structured hospital stewardship programs. Complementing these observations, the network meta‐analysis by Geng et al. [27] demonstrates that combined infection–prevention bundles, including contact precautions, chlorhexidine bathing, and enhanced environmental hygiene, are the most effective strategies for reducing MDR organisms acquisition. In summary, MDR pathogens pose a substantial challenge to the safety of surgical patients, particularly in healthcare settings with limited diagnostic resources and suboptimal stewardship practices. Strengthening AMR surveillance and implementing stratified antibiogram models are critical steps toward refining empirical therapy, mitigating AMR progression, and improving patient outcomes. Achieving this objective requires sustained collaboration between microbiology laboratories and antimicrobial stewardship programs [21, 22].

In this context, the present study aims to comprehensively evaluate the prevalence, microbial spectrum, resistance profiles, and clinical risk factors of pathogens responsible for nosocomial infections among surgical inpatients in a Romanian tertiary‐care general surgery unit.

## 2. Materials and Methods

### 2.1. Study Design and Setting

This retrospective observational study was conducted in Clinic III of the General Surgery Department at “St. Spiridon” Emergency Clinical Hospital, Iasi, Romania, a tertiary referral center affiliated with the “Grigore T. Popa” University of Medicine and Pharmacy. The study covered a 2‐year period from January 1, 2021, to December 31, 2022, spanning both the COVID‐19 pandemic and the subsequent postpandemic transition. This timeframe enabled the assessment of nosocomial infection dynamics and AMR trends under distinct epidemiological conditions.

### 2.2. Study Population

The study population comprised all patients admitted to Clinic III Surgery during the 24‐month period (2021–2022) who received antimicrobial treatment, either prophylactic (perioperative) or therapeutic (curative). A total of *n* = 2500 patients were initially reviewed, representing *n* = 1372 male and *n* = 1128 female. Following the exclusion of 29 (1.16%) due to missing essential clinical data for key variables, a total of 2471 remained in the final analysis. Therefore, the cohort consisted of *n* = 1360 (55.03%) men and *n* = 1111 (44.96%) women.

### 2.3. Data Collection and Structure

Patient‐level data were extracted from electronic medical records and microbiology laboratory reports and subsequently compiled into structured datasets. All consecutive patients admitted during the established interval were screened for eligibility. Inclusion criteria included (i) hospital admission between January 1, 2021 and December 31, 2022, (ii) to have at least one sample collected for microbiological analysis, (iii) receipt of antimicrobial therapy, either perioperative prophylaxis or curative treatment, and (iv) availability of microbiological investigations when nosocomial infection was suspected. Exclusion criteria included (i) incomplete clinical and (ii) microbiological data for key variables or (iii) isolates from the same patient. Patients under 18 years of age were not excluded a priori and were retained in the cohort if they met the above criteria.

The final dataset incorporated demographic variables (age, sex, and residential background [urban or rural]), along with clinical parameters (neoplasms, SARS‐CoV‐2 infection, perioperative antibioprophylaxis exposure, ICU admission, and total duration of hospitalization). Microbiological parameters included pathogen identification, susceptibility profiles, and classification of infections as MDR or non‐MDR, in accordance with European Committee on Antimicrobial Susceptibility Testing (EUCAST) clinical breakpoints and the Magiorakos et al. [28] criteria.

To address potential confounding factors, comorbidities commonly associated with infection risk or AMR, including diabetes mellitus, chronic kidney or liver disease, cardiovascular disease, obesity, immunosuppressive conditions, and neoplasms, were systematically extracted from medical records and incorporated into the multivariate regression models. Variables with established biological plausibility or demonstrated association in prior literature [29–31] were included a priori as covariates. Comorbidities with insufficient prevalence or inconsistent documentation were retained descriptively but excluded from regression analysis to avoid model instability.

Microbiological data were verified by clinical microbiologists before inclusion, ensuring standardized interpretation of culture results and susceptibility profiles.

The final dataset was structured at both patient and specimen level. A total of 99 (*n* = 3 non‐MDR [3.03%] vs. *n* = 96 MDR [96.96%]) patients presented with more than one infectious episode and/or more than one microbiological culture collected during hospitalization. In addition, *n* = 152 specimen cultures yielded polymicrobial growth (*n* = 5 non‐MDR [3.28%] vs. *n* = 147 MDR [96.71%]), with more than one bacterial species isolated from the same sample. Clinical specimens included wound secretions, wound slough, abscess material (purulent collections), tracheal secretions and aspirates, blood cultures, stool samples, bile, urine, peritoneal fluid, drainage fluid, catheter tips, nasopharyngeal exudates, puncture fluids, ascites, pleural fluid, and sputum.

### 2.4. Microbiological Testing and Resistance Classification

All microbiological investigations were performed in the hospital′s microbiology laboratory using standardized diagnostic protocols. Clinical, microbiological, and antibiotic utilization data were extracted from integrated hospital electronic medical records and laboratory information systems and linked at the individual patient level using unique admission identifiers, ensuring consistency and completeness across datasets. Antimicrobial susceptibility testing (AST) was carried out using the broth microdilution method with the MICRONAUT system (Merlin Diagnostika GmbH, Germany), and results were interpreted according to the EUCAST clinical breakpoints applicable at the time of isolate collection.

Aerobic pathogens were identified using routine culture techniques followed by standard phenotypic characterization and automated identification systems. Anaerobic bacteria were processed using dedicated culture techniques. Identification of anaerobic isolates was performed by matrix‐assisted laser desorption/ionization time‐of‐flight mass spectrometry (MALDI‐TOF MS; Bruker MALDI Biotyper). AST for anaerobic isolates was also performed using broth microdilution with the MICRONAUT system.

Infections caused by toxin‐producing anaerobic organisms were diagnosed by direct detection of specific toxins using immunochromatographic assays performed on stool specimens, in accordance with routine diagnostic algorithms. In these cases, culture‐based identification was not routinely performed.

Classification of isolates into susceptible (S), intermediate (I), or resistant (R) categories was based on EUCAST clinical breakpoints. MDR was defined following the internationally accepted criteria proposed by Magiorakos et al. [28], whereby an isolate was classified as MDR if it demonstrated nonsusceptibility to at least one agent in ≥ 3 antimicrobial categories. These definitions are consistent with those adopted by the ECDC. Patients whose isolates did not meet the MDR definition were classified as non‐MDR.

### 2.5. Data Analysis

All collected data were initially processed using Microsoft Excel 2010 (Microsoft Corporation, Redmond, WA, USA). Statistical analyses were conducted using GraphPad Prism version 9.1.0 (GraphPad Software, LLC, San Diego, CA, USA) and IBM SPSS Statistics version 26.0 (IBM Corp., Armonk, NY, USA). Statistical significance was set at a two‐sided *p* < 0.05.

Descriptive statistics were used to summarize demographic, clinical, and infection‐related characteristics. Continuous variables were assessed for normality using the Shapiro‐Wilk test and are presented as median with interquartile range (IQR), reflecting their non‐normal distribution. Categorical variables are summarized as frequencies and percentages. Between‐group comparisons (MDR vs. non‐MDR) were performed using the Fisher′s exact test for categorical variables and the Mann–Whitney *U* test for continuous variables.

Comorbidity burden was quantified as the total number of documented chronic conditions per patient and subsequently operationalized as a binary variable (≥ 2 vs. 1) to reflect overall clinical complexity. This variable was included as a confounder in the univariate and multivariate logistic regression analyses together with other clinically relevant covariates. Given the substantial overlap between individual comorbidities, this aggregated approach was adopted to minimize multicollinearity and reduce the risk of model overfitting.

A multivariate logistic regression model was constructed to identify independent predictors of MDR acquisition. Variables demonstrating an association with MDR status at *p* < 0.10 in univariate analyses or deemed clinically relevant were considered eligible for inclusion. Model selection followed a forward stepwise approach, with entry and retention thresholds of *p* < 0.10 and *p* < 0.05, respectively. Multicollinearity was assessed using the variance inflation factor (VIF), with values < 5 considered acceptable. Model fit and parsimony were evaluated using the Akaike Information Criterion (AIC) and Bayesian Information Criterion (BIC). Results are reported as adjusted ORs [aORs] with 95% confidence interval [95% CI].

ROC curve analysis was applied to assess the discriminative performance of selected variables for in‐hospital mortality. The area under the ROC curve (AUC), 95% CI, and *p* values were calculated to assess discriminative performance.

**Figure 1:**
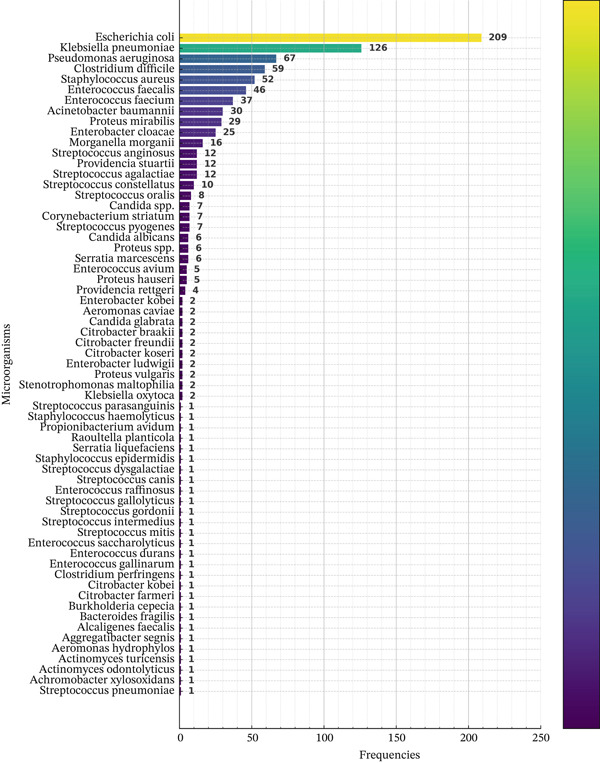
Descriptive distribution of bacterial and fungal species isolated from clinical samples from surgical inpatients included in the study cohort (2021–2022); no error bars or inferential statistical comparisons are shown. Bars show the absolute number of isolates/species occurrences, ordered from highest to lowest frequency; values at the end of each bar indicate counts. Because multiple specimens and polymicrobial cultures were recorded for some patients, frequencies represent isolate‐/specimen‐level occurrences rather than unique patients. The color gradient reflects relative frequency, with lighter colors indicating more frequently isolated microorganisms and darker colors indicating less frequent isolates.

**Figure 2:**
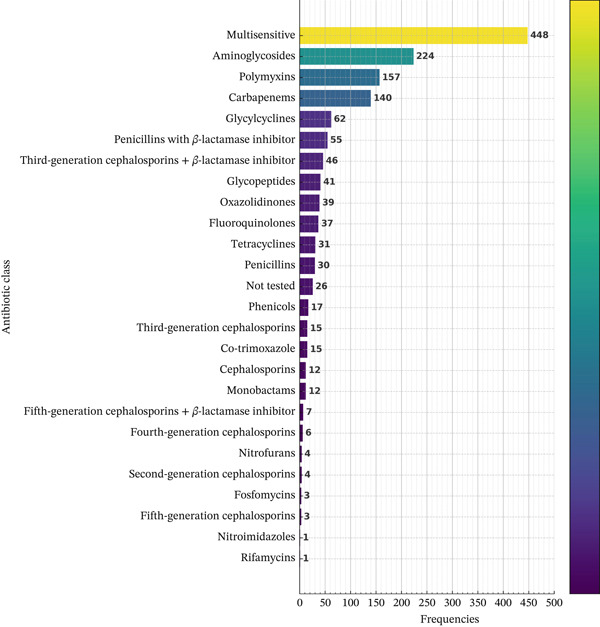
Descriptive distribution of antibiotic classes based on antimicrobial susceptibility profiles of bacterial and fungal isolates; no error bars or inferential statistical comparisons are shown. Bars represent the absolute frequency of isolates classified according to susceptibility to specific antibiotic classes, ordered from highest to lowest frequency; values at the end of each bar indicate counts. “Multisensitive” refers to isolates susceptible to multiple antimicrobial classes, whereas “Not tested” indicates that susceptibility to the respective class was not evaluated. Because multiple specimens and polymicrobial cultures were included, frequencies reflect isolate‐level occurrences rather than unique patients. The color gradient represents relative frequency, with lighter colors indicating more frequent categories and darker colors indicating less frequent ones.

Color‐graded horizontal bar charts showing the distribution of microbial species (Figure 1) and antibiotic classes (Figure 2) were created using Python (version 3.12; Python Software Foundation, USA) with the matplotlib (version 3.8.4) and pandas (version 2.2.2) libraries [32, 33].

### 2.6. Ethical Approval

This study was conducted in accordance with the ethical principles of the Declaration of Helsinki and applicable national and European regulations governing biomedical research. The study protocol was approved by the Ethics Committee of the “Grigore T. Popa” University of Medicine and Pharmacy, Iasi (no. 597/3.05.2025). The research was conducted in accordance with Romanian Law No. 206/2004 on good conduct in scientific research, technological development, and innovation, as well as relevant EU directives on research ethics. All patient data were fully anonymized prior to analysis, and no interventions or procedures beyond standard clinical care were performed.

## 3. Results

### 3.1. Patient Demographics and Clinical Characteristics

Patients with MDR infections exhibited significantly different clinical and demographic characteristics compared with those with non‐MDR infections (Table 1). Patients in the MDR group were significantly older, with a median age of 62 years (IQR: 51.75–71.25), compared with 54 years (IQR: 40–68) in the non‐MDR group (*p* < 0.0001). This age difference suggests a higher burden of MDR infections among older patients, potentially reflecting cumulative healthcare exposure, a greater prevalence of comorbidities, and age‐related immune vulnerability.

**Table 1:** Comparative analysis of demographic and clinical characteristics between MDR and non‐MDR patient groups.

Parameter	MDR (*n* = 370)	Non‐MDR (*n* = 2101)	*p*
Age (years)	62.0 (51.75–71.25)	54.0 (40.0–68.0)	< 0.0001
Gender (M/F)	248 (67.03%)/122 (32.97%)	1112 (52.93%)/989 (47.07%)	< 0.0001
Residence (R/U)	183 (49.46%)/187 (50.54%)	1027 (48.88%)/1074 (51.12%)	0.865
Hospitalization days	14.0 (9.0–23.0)	6.0 (4.0–9.0)	< 0.0001
ICU days	0 (0–3)	0 (0–0)	< 0.0001
COVID‐19 (N/P)	326 (88.11%)/44 (11.89%)	2022 (96.24%)/79 (3.76%)	< 0.0001
Neoplasms (B/M)	319 (86.22%)/51 (13.78%)	1883 (89.62%)/218 (10.38%)	0.057
Antibioprophylaxis (N/Y)	358 (96.76%)/12 (3.24%)	956 (45.50%)/1145 (54.50%)	< 0.0001
Comorbidities (1/≥ 2)	308 (83.24%)/62 (16.76%)	1882 (89.58%)/219 (10.42%)	0.0007

*Note:* Continuous variables are presented as median and IQR due to non‐normal distribution and were compared using the Mann–Whitney *U* test. Categorical variables are presented as number (percentage) and were compared using Fisher′s exact test. A two‐sided *p* value < 0.05 was considered statistically significant.

Hospitalization differed markedly between the two groups. The median length of hospital stay was significantly longer among the MDR group (14 days; IQR: 9–23) than among the non‐MDR group (6 days; IQR: 4–9; *p* < 0.0001). Similarly, ICU stays were higher in the MDR group, with a median of 0 days (IQR: 0–3), compared with 0 days (IQR: 0–0) in the non‐MDR group (*p* < 0.0001). These findings reflect the increased clinical complexity and healthcare resource utilization associated with MDR infections.

To facilitate data interpretation and provide a concise overview of MDR distribution, a comparative perspective illustrating MDR prevalence among ICU and non‐ICU patients is provided in Figure S1. This visualization highlights the markedly higher burden of MDR infections in the ICU setting, complementing the regression‐based findings.

Sex distribution also differed significantly between groups (*p* < 0.0001). In the MDR group, 67.03% of patients were male (*n* = 248) and 32.97% were female (*n* = 122), whereas the non‐MDR group showed a more balanced distribution (52.93% male [*n* = 1112] vs. 47.07% female [*n* = 989]). This male predominance among MDR cases may reflect sex‐related differences in exposure patterns, comorbidity profiles, or healthcare‐seeking behavior.

No significant differences were observed regarding place of residence (*p* = 0.865), with similar proportions of rural (*n* = 183 [49.46%]/*n* = 1027 [48.88%]) and urban (*n* = 187 [50.54%]/*n* = 1074 [51.12%]) patients in both MDR and non‐MDR groups being observed, suggesting that residential setting alone did not influence MDR acquisition in this cohort.

The prevalence of neoplasms did not differ significantly between groups, being reported in 13.78% (*n* = 51) of MDR patients and 10.38% (*n* = 218) of non‐MDR patients. Benign neoplasms were prevalent in both arms, accounting for 86.22% (*n* = 319) of MDR patients and 89.62% (*n* = 1883) of non‐MDR cases (*p* = 0.057). SARS‐CoV‐2 infection was significantly more frequent among MDR patients. Positive status was identified in 11.89% (*n* = 44) of MDR patients compared with 88.11% (*n* = 326) negative, respectively, 3.76% (*n* = 79) and 96.24% (*n* = 2022) in the non‐MDR group (*p* < 0.0001). This association may reflect increased antimicrobial exposure, prolonged hospitalization, or immune dysregulation in patients with recent or concurrent COVID‐19 infection.

A pronounced difference was observed in perioperative antibiotic prophylaxis. Only 3.24% (*n* = 12) of patients in the MDR group received prophylactic antibiotics, compared with 54.50% (*n* = 1145) in the non‐MDR group (*p* < 0.0001). Conversely, the absence of prophylaxis was substantially more common in the MDR group (96.76% [*n* = 358] vs. 45.50% [*n* = 956]). This inverse association highlights important differences in perioperative antimicrobial exposure between groups and underscores the potential role of appropriate antimicrobial stewardship in surgical settings.

Regarding comorbidity burden, patients with ≥ 2 comorbidities accounted for a significantly higher proportion of MDR infections compared with those with a single comorbidity (16.76% [*n* = 62] vs. 10.42% [*n* = 219]; *p* < 0.0007). In contrast, among non‐MDR patients, the majority had a single comorbidity (83.24% [*n* = 308] vs. 89.58% [*n* = 1882]). Overall, the presence of multiple comorbidities was associated with an increased likelihood of MDR infection, supporting the role of cumulative disease burden as a key risk factor for AMR.

### 3.2. In‐Hospital Mortality and Risk Factors

Overall, *n* = 154 out of *n* = 2471 patients (6.2%) died during hospitalization. Mortality differed significantly between the MDR and non‐MDR groups. Patients with MDR infections exhibited a markedly higher mortality rate compared with those without MDR infections (16.76% [*n* = 62/370] vs. 4.38% [*n* = 92/2101]; Fisher′s exact test, *p* < 0.0001). This association was further supported by effect size estimates, with a relative risk of 3.029 (95% CI: 2.412–3.732) and an odds ratio (ORs) of 4.396 (95% CI: 3.095–6.214), indicating a substantially increased risk of death among MDR patients.

To further evaluate independent predictors of mortality, a multivariate logistic regression model was constructed, including age, length of hospitalization, ICU stay, and MDR status. The overall model was statistically significant (omnibus *χ* ^2^ = 389.327, df = 4, *p* < 0.001) and demonstrated moderate explanatory power (Nagelkerke *R* ^2^ = 0.391).

Within the multivariate model, MDR infection remained an independent predictor of in‐hospital mortality (aOR = 3.080, 95% CI: 1.813–5.232, *p* < 0.001). ICU stay was identified as the strongest predictor, with each additional day associated with a 46.4% increase in the odds of death (aOR = 1.464, 95% CI: 1.377–1.556, *p* < 0.001), highlighting the critical impact of disease severity.

Age was also independently associated with mortality, with each additional year increasing the odds of death by approximately 5.7% (aOR = 1.057, 95% CI: 1.043–1.072, *p* < 0.001). In contrast, length of hospitalization was inversely associated with mortality (aOR = 0.849, 95% CI: 0.810–0.891, *p* < 0.001), likely reflecting early mortality among more severe cases rather than a protective effect of prolonged hospitalization.

The model demonstrated high overall classification accuracy (95.4%), although sensitivity for predicting mortality remained limited (35.7%), indicating reduced performance in identifying fatal outcomes.

### 3.3. Microbial Spectrum and Resistance Profiles

The microbiological analysis revealed a broad and heterogeneous spectrum of pathogens, with several dominant organisms of major clinical relevance for empirical antimicrobial selection and infection control strategies (Figure 1).

*E. coli* was the most frequently isolated pathogen (*n* = 209), confirming its pivotal role in postoperative infections, particularly those involving the urinary and intra‐abdominal compartments. *K. pneumoniae* ranked second in prevalence (*n* = 126), highlighting its prominence as a prominent nosocomial pathogen and a well‐recognized reservoir of MDR mechanisms, including ESBLs and carbapenemases.

Nonfermenting Gram‐negative bacilli were also commonly identified, with *P. aeruginosa* (*n* = 67) and *A. baumannii* (*n* = 30) representing a substantial proportion of isolates. These organisms are frequently associated with healthcare‐ and ICU‐related infections and are characterized by intrinsic resistance and a marked capacity to acquire additional AMR determinants.

Among Gram‐positive organisms, *Clostridium difficile* was frequently detected (*n* = 59), likely reflecting extensive antibiotic exposure and subsequent disruption of intestinal microbiota in the surgical population. *S. aureus* (*n* = 52), including MRSA, and *Enterococcus* species, particularly *Enterococcus faecalis* (*n* = 46) and *E. faecium* (*n* = 37), were also prominent, underscoring their established roles in SSI, BSI, and device‐associated infections.

Beyond the most prevalent pathogens, the microbiological landscape demonstrated considerable diversity. More than 50 additional bacterial species were isolated at lower frequencies (ranging from 1 to 29 isolates per species), including opportunistic and less commonly encountered organisms, such as *Raoultella planticola*, *Actinomyces* spp., and *Bacteroides fragilis*. This wide microbial heterogeneity reflects the complex and often polymicrobial nature of infections in surgical patients and emphasizes the necessity of continuous microbiological surveillance to detect emerging pathogens and evolving resistance patterns.

### 3.4. Antibiotic Utilization Patterns

A key observation was the high proportion of multisusceptible isolates (*n* = 448), indicating that a considerable fraction of infections remained susceptible to multiple antimicrobial classes (Figure 2). While this finding is encouraging, it may also reflect a predominance of early‐onset or community‐acquired infections, where prior exposure to broad‐spectrum antibiotics and selective pressure are comparatively limited.

Among antibiotic classes demonstrating the highest recorded activity, aminoglycosides were most frequently effective (*n* = 224), followed by polymyxins (*n* = 157) and carbapenems (*n* = 140). These results highlight the continued reliance on broad‐spectrum and last‐resort agents, particularly in the management of infections caused by MDR Gram‐negative organisms. Such patterns likely reflect both the severity of infections encountered and the restricted therapeutic options available in resistant settings.

Moderate activity was observed with glycylcyclines, glycopeptides, and oxazolidinones, agents predominantly reserved for resistant Gram‐positive infections, including MRSA and vancomycin‐resistant *Enterococcus* species. In contrast, older or narrower‐spectrum agents such as penicillins, tetracyclines, and phenicols were used less frequently, likely due to reduced efficacy in severe nosocomial infections and widespread resistance.

A small subset of isolates (*n* = 26) was categorized as “not tested,” potentially reflecting logistical constraints, incomplete laboratory investigations, or the initiation of empirical therapy in the absence of microbiological confirmation. These gaps underscore the importance of comprehensive susceptibility testing to support rational antimicrobial selection and stewardship efforts.

Comparative analysis of antibiotic utilization between MDR patients and non‐MDR revealed distinct patterns in both frequency and duration of administration across pharmacological classes (Table 2). The most commonly used agents in both cohorts were aminopenicillins with *β*‐lactamase inhibitors, administered in *n* = 182 MDR cases (median duration: 5.5 [3–7] days) and *n* = 675 non‐MDR cases (4 [3–6] days), reflecting their central role in empirical and targeted therapy irrespective of resistance profile. Aminopenicillins without *β*‐lactamase inhibitors were used infrequently in both groups, but were associated with longer treatment durations among MDR patients (*n* = 17 cases, 7; [5–10] days) compared with non‐MDR patients (*n* = 7 cases, 2; [2–4.5] days). This pattern suggests limited effectiveness of narrow‐spectrum aminopenicillins in resistant infections and reflects their restricted use, primarily in selected clinical contexts or de‐escalation strategies. Classical penicillins were prescribed at low frequencies, with *n* = 11 MDR cases and *n* = 7 non‐MDR cases. Treatment duration tended to be slightly longer in MDR patients (*n* = 7 [6–9.5] vs. *n* = 5 [3.5–9]), indicating cautious and highly selective use, likely restricted to susceptible isolates or specific indications.

**Table 2:** Comparative analysis of antibiotic class utilization among MDR and non‐MDR surgical patients: median duration of administration and intraoperative/perioperative frequencies.

Antibiotic class	*n*, median, IQR (MDR)	*n*, median IQR (non‐MDR)	Periop MDR (*n*)	Intraop MDR (*n*)	Periop non‐MDR (*n*)	Intraop non‐MDR (*n*)
Aminoglycosides	85, 5 (4–8)	16, 6 (4–7.5)	2	2	8	4
Aminopenicillin + *β*‐lactamase inhibitor	182, 5.5 (3–7)	675, 4 (3–6)	3	14	326	10
Aminopenicillins	17, 7 (5–10)	7, 2 (2–4.5)	—	—	2	2
Carbapenem + dehydropeptidase inhibitor	10, 6.5 (2.25–9)	7, 4 (3.5–5.5)	—	—	—	—
Carbapenems	86, 26 (5–9)	56, 5 (3–5.25)	1	1	3	2
Classical penicillins	11, 7 (6–9.5)	7, 5 (3.5–9)	—	—	—	—
Fourth‐generation cephalosporin	5, 7 (4–7)	—	—	—	—	—
Fourth‐generation fluoroquinolone	9, 7 (2–7)	5, 7 (5–7)	—	1	—	—
Glycopeptides	86, 8 (6–10)	57, 6 (4–8)	1	1	2	2
Glycylcycline	17, 8 (5–10)	1, 6 (N/A)	—	—	—	—
Lincosamides	11, 3 (2.5–5.5)	11, 7 (4–8.5)	—	—	3	—
Macrolides	2, 5 (4–6)	5, 4 (3–5)	—	—	—	—
Nitroimidazoles	162, 6 (4–8)	465, 4 (3–6)	2	1	60	—
Oxazolidinones	23, 8 (6.5–10)	6, 6.5 (5.25–7)	—	—	1	—
Penicillinase‐resistant penicillin	9, 8 (6–9)	3, 4 (3–4)	—	—	2	1
Penicillins with *β*‐lactamase inhibitors	23, 7 (3–9)	17, 6 (4–7)	—	—	2	—
Polymyxins	50, 7.5 (5–10)	5, 4 (4–7)	—	—	—	—
Rifamycins	6, 6.5 (5.25–7.75)	—	—	—	—	—
Second‐generation cephalosporins	16, 4 (3–7.75)	72, 3 (3–5)	10	25	665	—
Second‐generation fluoroquinolone	84, 5 (3–7.75)	85, 4 (3–5)	2	1	16	1
Sulfonamides and folate synthesis inhibitors	1, 10 (N/A)	2, 3.5 (2.75–4.25)	—	—	—	—
Tetracycline	1, 7 (N/A)	—	—	—	—	—
Third‐generation cephalosporin	101, 6 (4–8)	146, 4 (3–6)	4	2	89	28
Third‐generation cephalosporin + *β*‐lactamase inhibitor	3, 2 (2–7.75)	3, 6 (6.6.5)	—	—	—	—
Third‐generation fluoroquinolone	22, 5.5 (4.25–10)	8, 5 (2.75–7.5)	—	—	1	—

Nitroimidazoles were also frequently prescribed, particularly in the non‐MDR group (*n* = 465 vs. *n* = 162), although treatment duration was longer in MDR patients (*n* = 6 [4–8] vs. *n* = 4 [3–6] days), likely due to increased infection complexity and anaerobic involvement.

Carbapenems (*n* = 86 MDR [26 (5–9)] vs. *n* = 56 non‐MDR [5 (3–5.25)] cases) and their dehydropeptidase inhibitor combinations (*n* = 10 [6.5 (2.25–9)] vs. *n* = 7 [4 (3.5–5.5)] cases) were associated with significantly prolonged courses in MDR patients, reinforcing their reserved use for resistant Gram‐negative infections. Similarly, glycopeptides and oxazolidinones were both more frequent and administered for longer durations in MDR cases (glycopeptides: *n* = 86 cases, 8 [6–10] days; oxazolidinones: *n* = 23 cases, 8 [6.5–10] days) compared to *n* = 57 (6 [4–8]), respectively, *n* = 6 (6.5 [5.25–7]) cases in the non‐MDR group. Glycylcyclines, used almost exclusively in MDR settings (*n* = 17 vs. *n* = 1 case), were associated with some of the longest treatment durations ([8 (5–10)] days).

Among broad‐spectrum agents, aminoglycosides were used in *n* = 85 MDR and *n* = 16 non‐MDR cases, with comparable treatment durations (5 [4–8] vs. 6 [4–7.5]), reflecting their adjunctive role in severe infections. Third‐generation cephalosporins were more frequently prescribed in non‐MDR patients (*n* = 146 vs. *n* = 101) but were administered for longer durations in MDR contexts (6 [4–8] vs. 4 [3–6] days). A similar trend was observed for second‐generation cephalosporins, predominantly used in non‐MDR infections (*n* = 72 vs. *n* = 16) but with prolonged exposure among MDR patients (4 [3–7.75] vs. 3 [3–5] days). Fourth‐generation cephalosporins were rarely employed and exclusively observed in the MDR cohort (*n* = 5 cases, 7 [4–7]), reflecting their role as reserve agents in severe infections caused by resistant Gram‐negative organisms. Their complete absence in the non‐MDR group further underscores stewardship‐driven restriction of advanced‐generation cephalosporins.

Fluoroquinolones use demonstrated nuanced patterns across generations: second‐generation agents were nearly equally represented (*n* = 84 MDR vs. *n* = 85 non‐MDR), though treatment lasted longer in MDR cases (5 [3–7.75] vs. 4 [3–5] days). Third‐generation fluoroquinolones followed a similar trend (*n* = 22 MDR vs. *n* = 8 non‐MDR; 5.5 [4.25–10] vs. 5 [2.75–7.5] days). Fourth‐generation fluoroquinolones, while rare, showed reverse patterns, being slightly more prolonged prescribed in non‐MDR patients (*n* = 5; 7 [5–7] vs. *n* = 9; 7 [2–7] days). Classes with restricted usage in both groups included macrolides, tetracyclines, sulfonamides, rifamycins, and third‐generation cephalosporins with *β*‐lactamase inhibitors with low frequencies (≤ 6) and variable durations.

Polymyxins, representing last‐line therapy for extensively resistant Gram‐negative pathogens, were administered almost exclusively in *n* = 50 MDR patients (7.5 [5–10] days) vs. only *n* = 5 non‐MDR cases (4 [4–7] days), indicating their near‐exclusive use in MDR scenarios. Interestingly, lincosamides were prescribed with similar frequency in the non‐MDR group (*n* = 11 vs. *n* = 11) but were administered for a significantly longer duration in non‐MDR (7 [4–8.5] vs. 3 [2.5–5.5] days), potentially reflecting extended perioperative or anaerobic coverage in less resistant infections. In contrast, penicillinase‐resistant penicillins and penicillins + *β*‐lactamase inhibitors were used for longer durations among MDR patients (9 [8 (6–9)]/23 [7 (3–9)]).

### 3.5. Perioperative and Intraoperative Antibiotic Administration

Marked differences were observed in the selection and frequency of antibiotics administered perioperatively between MDR and non‐MDR patient cohorts, reflecting divergent prophylactic strategies shaped by anticipated resistance profiles and antimicrobial stewardship considerations.

Among non‐MDR patients, second‐generation cephalosporins were by far the most frequently administered perioperative agents (*n* = 665), reaffirming their central role in standard surgical prophylaxis due to their favorable safety profile and balanced antimicrobial spectrum. In contrast, this class was used in only *n* = 10 perioperative MDR cases, indicating a markedly more restrictive application in the context of resistant infections. Aminopenicillin with *β*‐lactamase inhibitors represented the second most common perioperative regimen in non‐MDR cases (*n* = 326), likely reflecting extended prophylaxis in procedures associated with increased complexity or comorbidity burden. Conversely, this combination was administered perioperatively in only *n* = 3 MDR cases, underscoring a conservative approach when resistance was anticipated.

Third‐generation cephalosporins were used perioperatively in *n* = 89 non‐MDR cases but in only *n* = 4 MDR patients, suggesting a deliberate shift away from conventional broad‐spectrum prophylaxis toward more selective strategies in MDR contexts. Nitroimidazoles, typically employed for anaerobic coverage in colorectal and gynecological surgery, were administered *n* = 60 times in non‐MDR patients but only twice among MDR cases, indicating a preferential reservation for postoperative or therapeutic use rather than routine prophylaxis in resistant contexts. Aminoglycosides were rarely used perioperatively in either groups (*n* = 8 non‐MDR vs. *n* = 2 MDR cases), supporting their selective deployment in high‐risk scenarios rather than as standard prophylaxis regimens.

Other antibiotic classes were only sporadically administered perioperatively. Glycopeptides were recorded in *n* = 3 cases overall, *n* = 2 non‐MDR and *n* = 1 MDR, likely reflecting targeted Gram‐positive or MRSA coverage. Lincosamides, including clindamycin, appeared exclusively in *n* = 3 non‐MDR cases, consistent with their role in penicillin‐allergic patients, while oxazolidinones, a last‐line class for resistant Gram‐positive infections, were administered only once and exclusively in the non‐MDR group. Penicillinase‐resistant penicillins and penicillin with *β*‐lactamase inhibitors combinations were each used in *n* = 2 non‐MDR patients each and were completely absent in the MDR perioperative group. Notably, no perioperative use of macrolides, polymyxins, rifamycins, fourth‐generation cephalosporins, fourth‐generation fluoroquinolones, glycylcyclines, tetracyclines, or sulfonamides were observed in either cohort, underscoring strict adherence to stewardship principles and reserving these agents for therapeutic indication.

Overall, perioperative administration of less commonly used antibiotic classes was limited in both cohorts, reinforcing a cautious and stewardship‐oriented prophylactic strategy. Among non‐MDR patients, narrow‐spectrum aminopenicillins were rarely employed perioperatively (*n* = 2), reflecting their limited role in surgical prophylaxis due to insufficient Gram‐negative coverage. Carbapenems were used in only three non‐MDR cases and one MDR, indicating exceptional circumstances rather than routine practice. Second‐generation fluoroquinolones were administered perioperatively in *n* = 16 non‐MDR patients and two MDR, suggesting selective use in specific clinical scenarios, while third‐generation fluoroquinolones were recorded in a single non‐MDR case. In contrast, perioperative exposure to these agents in the MDR cohort was even more restricted: aminopenicillins, classical penicillins, carbapenem‐dehydropeptidase inhibitor combinations, third‐generation fluoroquinolones, and third‐generation cephalosporin/*β*‐lactamase inhibitor combinations were entirely absent, while carbapenems and second‐generation fluoroquinolones were recorded in only one and two MDR cases, respectively. Together, these findings illustrate a markedly more conservative perioperative antibiotic approach in MDR patients, characterized by stringent avoidance of broad‐spectrum and last‐line agents.

Intraoperative antibiotic utilization also differed substantially between MDR and non‐MDR patients. Among MDR cases, second‐generation cephalosporins were the most frequently administered intraoperative agents (*n* = 25), highlighting their continued utility for immediate Gram‐positive and moderate Gram‐negative coverage even in high‐risk settings. In contrast, this class was not recorded intraoperatively among non‐MDR patients, where its use appeared confined primarily to the perioperative period. Aminopenicillin + *β*‐lactamase inhibitor combinations were administered 14 times intraoperatively in MDR cases, compared to *n* = 10 non‐MDR cases, reflecting their role in broad‐spectrum coverage when clinically indicated. Narrow‐spectrum aminopenicillins were rarely used intraoperatively, with no MDR cases and just *n* = 2 non‐MDR.

Notably, polymyxins and tetracyclines, agents typically reserved for highly resistant organisms were not administered intraoperatively in either group, suggesting that these antibiotics were either reserved for postoperative therapeutic use or deliberately avoided in the operating settings in accordance with antimicrobial stewardship policies.

Third‐generation cephalosporins were the most frequently used intraoperative agents in non‐MDR patients (*n* = 28), compared with only *n* = 2 MDR cases, suggesting a preferential optimization of Gram‐negative coverage in susceptible infections without escalation to reserve agents. Other antibiotic classes, including glycopeptides, rifamycins, lincosamides, and oxazolidinones, were used at very low frequencies, likely reflecting targeted coverage for resistant Gram‐positive pathogens. None of these agents, excepting glycopeptides, were administered intraoperatively in non‐MDR patients, further underscoring their selective use.

Similarly, fourth‐generation cephalosporins, macrolides, sulfonamides, and fourth‐generation fluoroquinolones just once in a MDR case were not utilized intraoperatively, suggesting adherence to strict intraoperative stewardship policies and reserving these agents for therapeutic rather than prophylactic indications. Overall, intraoperative antibiotic administration in MDR patients favored second‐generation cephalosporins and *β*‐lactam/*β*‐lactamase inhibitor combinations, whereas non‐MDR patients more frequently received third‐generation cephalosporins, likely reflecting risk‐adapted intraoperative decision‐making. The selective reservation of agents such as glycopeptides and oxazolidinones in MDR cases highlights a cautious, pathogen‐directed intraoperative approach in this subgroup.

Intraoperative administration of less frequently used antibiotic classes was limited in both non‐MDR and MDR cohorts, reflecting a restrictive and indication‐driven approach within the operating landscape. Among non‐MDR patients, aminoglycosides were administered intraoperatively in four cases, indicating selective use in specific high‐risk situations rather than routine coverage. Carbapenems were recorded in only two non‐MDR cases, underscoring their exceptional role and avoidance as standard intraoperative agents. Second‐generation fluoroquinolones and penicillinase‐resistant penicillins were each used once intraoperatively, highlighting sporadic, case‐specific utilization. Importantly, no intraoperative use of carbapenem‐dehydropeptidase inhibitor combinations, lincosamides, classical penicillins, glycylcyclines, penicillinase‐resistant penicillin/penicillins with *β*‐lactamase inhibitors, or third‐generation cephalosporin/fluoroquinolone/*β*‐lactamase inhibitor combinations was observed in the non‐MDR group.

In the MDR cohort, intraoperative exposure to these antibiotic classes was even more limited. Aminoglycosides were administered in only two MDR cases, while carbapenems and second‐generation fluoroquinolone were used intraoperatively in a single patient, reflecting their highly selective, last‐resort application. Nitroimidazoles were recorded in one MDR case, likely corresponding to targeted anaerobic coverage when clinically indicated. Notably, no intraoperative administration of carbapenem‐dehydropeptidase inhibitor combinations, classical penicillins, glycylcyclines, penicillinase‐resistant penicillins, penicillins with *β*‐lactamase inhibitors, or third‐generation cephalosporin/*β*‐lactamase inhibitor combinations or third‐generation fluoroquinolone was documented among MDR patients. Overall, these findings emphasize an even more stringent intraoperative stewardship strategy in MDR cases, characterized by the near‐complete avoidance of broad‐spectrum and reserve antibiotics and reliance on highly selective, indication‐driven administration.

### 3.6. Correlations Between Clinical Variables

**Figure 3:**
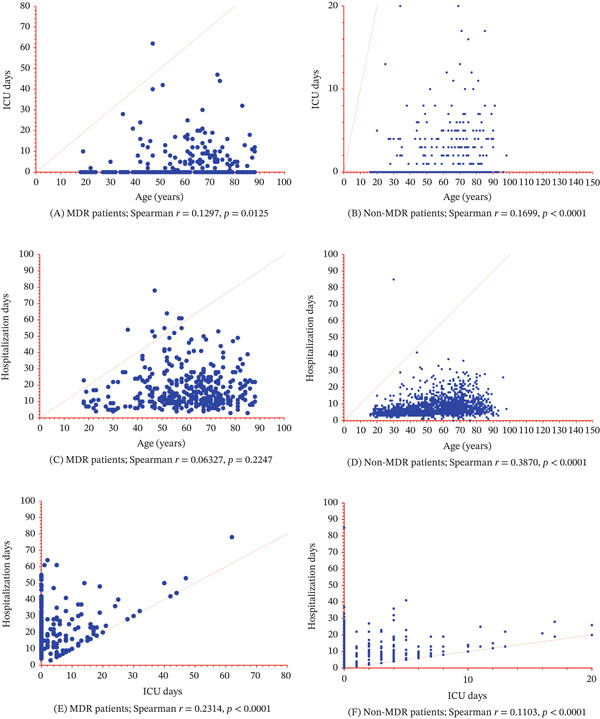
Spearman correlation analyses between age, ICU length of stay, and total hospitalization duration in patients with MDR and non‐MDR infections. Panels (A–F) illustrate pairwise relationships between clinical variables: (A) age versus ICU length of stay in MDR patients; (B) age versus ICU length of stay in non‐MDR patients; (C) age versus total hospitalization duration in MDR patients; (D) age versus total hospitalization duration in non‐MDR patients; (E) ICU length of stay versus total hospitalization duration in MDR patients; and (F) ICU length of stay versus total hospitalization duration in non‐MDR patients. Each point represents an individual patient. Spearman correlation coefficients (*r*) and corresponding *p* values are displayed in each panel. Dashed lines represent fitted trend lines. Statistically significant correlations (*p* = 0.0125, *p* < 0.0001) were observed between age and ICU stay in both cohorts, while age was not significantly associated with total hospitalization in MDR patients (*p* = 0.2247) but showed a moderate positive correlation in non‐MDR patients (*p* < 0.0001). ICU length of stay was positively associated with total hospitalization duration in both groups, with a slightly stronger correlation in MDR patients (*p* < 0.0001).

Correlation analyses demonstrated a weak but statistically significant positive association between patient age and ICU length of stay within the MDR cohort (*r* = 0.1297, *p* = 0.0125; Figure 3A), indicating that older patients with MDR infections tend to experience slightly longer ICU stays, although the magnitude of this association was limited. A similarly weak positive association was observed in the non‐MDR cohort (*r* = 0.1699, *p* < 0.0001; Figure 3B). Despite reaching statistical significance in both groups, the small effect size and substantial dispersion of data points suggest that age alone has a limited influence on ICU hospitalization duration, with other clinical and procedural factors likely playing a more prominent role.

When examined in relation to total hospitalization duration, the association with age showed greater variability across cohorts, suggesting context‐dependent effects. Within the MDR group, no statistically significant association was observed between patient age and overall length of hospitalization (*r* = 0.06327, *p* = 0.2247; Figure 3C), indicating that age was not meaningfully related to total inpatient stay among patients with MDR infections. In contrast, in the non‐MDR cohort, a moderate and statistically significant positive correlation was identified between age and total hospitalization length (*r* = 0.3870, *p* < 0.0001; Figure 3D). This finding suggests that, in the absence of AMR, increasing age is associated with longer hospital stays, likely reflecting age‐related vulnerability and comorbidity burden rather than resistance‐driven factors.

Importantly, weak but statistically significant positive correlations were observed between ICU length of stay and total hospitalization duration in both cohorts, underscoring the contribution of intensive care to overall inpatient time. This association was more pronounced among MDR patients (*r* = 0.2314, *p* < 0.0001; Figure 3E) compared with non‐MDR patients (*r* = 0.1103, *p* < 0.0001; Figure 3F). Although the effect sizes were modest, prolonged ICU stays were consistently associated with longer total hospitalization, with the relatively higher correlation observed in MDR patients likely reflecting greater clinical complexity and more protracted recovery trajectories associated with resistant infections.

### 3.7. Prognostic Prediction of Mortality

**Figure 4:**
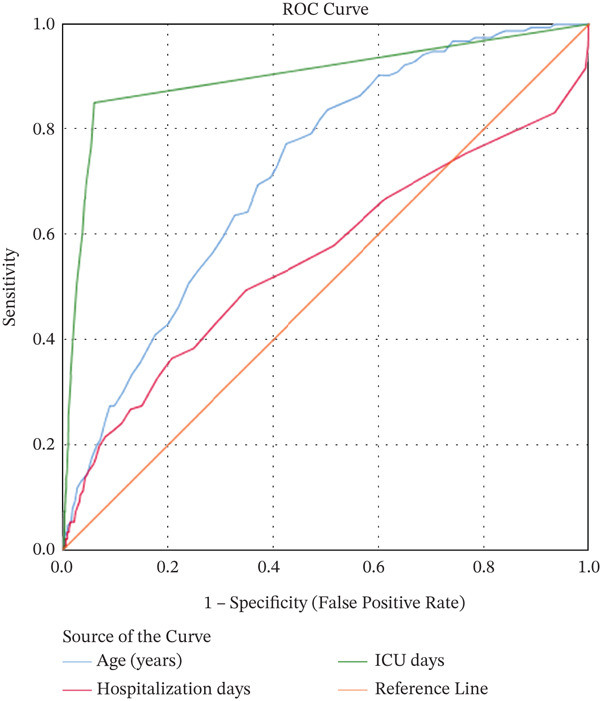
ROC curve analysis evaluating the predictive performance of age, total hospitalization duration, and ICU length of stay for in‐hospital mortality. ICU length of stay demonstrated the highest discriminative ability (AUC = 0.901; 95% CI: 0.867–0.934), followed by age (AUC = 0.721; 95% CI: 0.684–0.758), while total hospitalization duration showed limited predictive value (AUC = 0.555; 95% CI: 0.499–0.611). The diagonal reference line represents random classification (AUC = 0.5). These findings indicate that prolonged ICU stay is the strongest predictor of mortality, whereas overall hospitalization duration has minimal discriminatory capacity.

To assess the prognostic performance of key clinical variables for in‐hospital mortality, ROC curve analyses were conducted for age, total hospitalization duration, and ICU length of stay (Figure 4). Among the evaluated predictors, ICU length of stay showed the strongest discriminative ability, with an AUC of 0.901 (95% CI: 0.867–0.934;*p* < 0.0001), indicating excellent accuracy for distinguishing nonsurvivors from survivors. Age also demonstrated significant prognostic value, albeit more modest, with an AUC = 0.721; 95% CI: 0.684–0.758; *p* < 0.0001, consistent with fair discrimination. By contrast, total hospitalization duration displayed limited predictive utility (AUC = 0.555; 95% CI: 0.499–0.611; *p* = 0.022), reflecting only a marginal improvement over chance‐level classification.

Overall, these findings indicate that prolonged ICU stay is the most robust marker of adverse in‐hospital outcome in this surgical cohort, likely capturing the intensity of clinical deterioration. While age retains moderate prognostic relevance, overall hospitalization duration appears to be a weak discriminator between nonsurvivors and survivors, potentially influenced by nonmortality‐related factors such as discharge logistics, rehabilitation requirements, or prolonged management of complications that do not necessarily culminate in death. Notably, the ROC analysis identified at least one tie between positive and negative outcome groups for the tested variables, which may introduce minor bias into the nonparametric estimates; nevertheless, the overall pattern of discrimination, particularly the strong performance of ICU length of stay, remains consistent.

### 3.8. Multivariate Analysis of MDR Risk Factors

**Figure 5:**
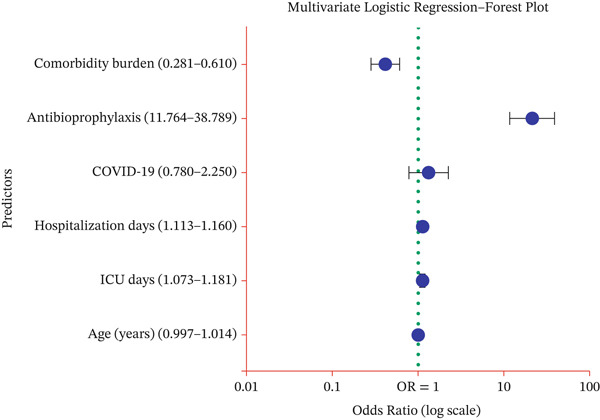
Forest plot of multivariate logistic regression analysis identifying independent predictors of MDR infection. aORs are displayed on a logarithmic scale, with horizontal lines representing 95% CIs. The vertical reference line at OR = 1 indicates no association. ICU length of stay (aOR = 1.126; 95% CI: 1.073–1.181) and total hospitalization duration (aOR = 1.136; 95% CI: 1.113–1.160) were independently associated with increased odds of MDR infection. Surgical antibiotic prophylaxis showed a strong positive association (aOR = 21.361; 95% CI: 11.764–38.789), likely reflecting confounding by indication. In contrast, age (aOR = 1.005; 95% CI: 0.997–1.013) and COVID‐19 status (aOR = 1.325; 95% CI: 0.780–2.250) were not significant predictors. Comorbidity burden was inversely associated with MDR risk (aOR = 0.414; 95% CI: 0.281–0.610), which may reflect differences in clinical pathways, patient selection, or residual confounding rather than a true protective effect.

A multivariate binary logistic regression was performed to identify independent predictors of MDR infections among *n* = 2471 patients (Figure 5). The model was statistically significant (omnibus *χ* ^2^ = 756.749, df = 6, *p* < 0.001), with a Nagelkerke *R* ^2^ value of 0.463, indicating that approximately 46.3% of the variance in MDR status was explained by the model. The overall classification accuracy was 87.5%, with high specificity for non‐MDR cases (96.7%) and moderate sensitivity for MDR cases (35.4%), reflecting the imbalance between MDR and non‐MDR cases.

Significant independent predictors of MDR infection included ICU length of stay, total hospitalization duration, surgical antibiotic prophylaxis, and comorbidity burden. Each additional day spent in the ICU was associated with an increase in the odds of MDR infection (aOR = 1.126, 95% CI: 1.073–1.181,*p* < 0.0001), while each additional hospitalization day increased MDR risk (aOR = 1.136, 95% CI: 1.113–1.160,*p* < 0.0001), underscoring the cumulative effect of prolonged healthcare exposure.

Antibiotic prophylaxis was strongly associated with MDR status (aOR = 21.361, 95% CI: 11.764–38.789,*p* < 0.0001). This association likely reflects confounding by indication, as prophylactic antibiotics are more frequently administered to patients undergoing complex surgical procedures or presenting with higher baseline infection risk, rather than implying a direct causal relationship.

Comorbidity burden also remained independently associated with MDR acquisition. Compared with patients presenting a single comorbidity, those with two or more comorbidities exhibited significantly lower adjusted odds of MDR infection (aOR = 0.414, 95% CI: 0.281–0.610,*p* < 0.0001), suggesting that comorbidity burden functions as a marker of distinct clinical pathways and patterns of care rather than a direct biological determinant of resistance.

In contrast, patient age (aOR = 1.005, 95% CI: 0.997–1.013,*p* = 0.201) and history of COVID‐19 (aOR = 1.325, 95% CI: 0.780–2.250,*p* = 0.298) were not independently associated with MDR infection after multivariate adjustment. Bootstrap validation using 1000 samples yielded consistent bias‐corrected and accelerated (BCa) CIs, confirming the robustness of the identified predictors.

Multicollinearity was assessed using VIF derived from auxiliary linear regression models including the same set of covariates. All VIF values were close to unity and well below the predefined threshold of 5, indicating no evidence of multicollinearity. Model fit and parsimony were evaluated using information criteria. The final multivariate model yielded an AIC of 1343.7 and a BIC of 1384.4, supporting the adequacy and parsimony of the selected model.

## 4. Discussion

This study, conducted in a Romanian tertiary‐care general surgery unit, identifies key clinical, microbiological, and treatment‐related characteristics associated with MDR infections. While broadly aligned with national and international trends, our findings provide specific insights applicable to surgical settings in Eastern Europe.

During the study period, no formal prospective antimicrobial stewardship intervention was implemented in the surgical unit beyond routine institutional policies. Perioperative prophylaxis followed standardized surgical protocols aligned with national recommendations, while therapeutic antibiotic choices were guided by routine microbiological reporting and EUCAST‐based susceptibility interpretation, with case‐by‐case input from clinical microbiologists or infectious disease specialists when MDR infection was suspected. Consequently, the observed differences in perioperative and intraoperative antibiotic selection, as well as the longer exposure to broad‐spectrum and reserve agents among MDR patients, likely reflect real‐world prescribing practices shaped by case‐mix complexity rather than the effect of a targeted stewardship intervention. Notably, the restrictive use of last‐line agents in perioperative and intraoperative settings suggests an underlying stewardship‐oriented prescribing culture. Together, these findings provide a pragmatic overview of antibiotic utilization under standard institutional stewardship constraints and underscore the need for future structured antimicrobial stewardship programs to better align prophylactic and therapeutic strategies with local resistance epidemiology and reduce avoidable selection pressure.

Patient‐related factors were prominent among MDR cases in unadjusted comparisons. Patients with MDR infections were significantly older than non‐MDR counterparts (median age: 62 years [IQR: 51.75–71.25] vs. 54 years [IQR: 40–68], *p* < 0.0001), consistent with established evidence linking advanced age to increased vulnerability to resistant infections through immunosenescence, cumulative healthcare exposure, and higher comorbidity burden [34, 35]. However, in multivariate analysis, age was not independently associated with MDR status (aOR = 1.005, 95% CI: 0.997–1.013, *p* = 0.201), suggesting that the observed age effect in bivariate analyses may be mediated by hospitalization‐related exposures and illness severity rather than age per se. This interpretation is supported by correlation analyses showing only weak associations between age and ICU length of stay in both cohorts (MDR: *r* = 0.1297, *p* = 0.0125; non‐MDR: *r* = 0.1699, *p* < 0.0001), and a cohort‐dependent relationship between age and total hospitalization, nonsignificant in MDR (*r* = 0.06327, *p* = 0.2247) but moderate in non‐MDR patients (*r* = 0.3870, *p* < 0.0001). Collectively, these findings indicate that age alone has a limited influence of ICU admission and overall length of stay once other clinical drivers are considered, in line with age‐stratified resistance trends reported in previous published studies [34, 35].

The present study demonstrates that MDR infections are independently associated with increased in‐hospital mortality. Even after adjustment for age, ICU stay, and length of hospitalization, MDR patients had more than a threefold higher risk of death. This finding highlights the clinical impact of AMR beyond disease severity alone. However, ICU stay emerged as the strongest predictor of mortality, suggesting that the severity of illness and need for intensive care play a central role in patient outcomes. Age was also independently associated with mortality, consistent with previous studies showing increased vulnerability among elderly patients. Interestingly, length of hospitalization was inversely associated with mortality. This likely reflects the fact that patients with severe disease tend to die earlier during hospitalization, rather than indicating a protective effect of longer hospital stay. Overall, these results suggest that while MDR infection is a significant independent risk factor, its impact is closely intertwined with critical illness and patient frailty. These findings are consistent with the growing global burden of AMR, which has been associated with increased mortality, prolonged hospitalization, and limited therapeutic options. Recent evidence indicates that MDR pathogens contribute substantially to adverse clinical outcomes, particularly in hospitalized patients, where resistance mechanisms such as plasmid‐mediated gene transfer and integron‐associated resistance facilitate rapid dissemination of MDR determinants [36].

Hospitalization‐related variables emerged as the strongest and most consistent correlates of MDR infection. The strong association between ICU exposure and MDR infection observed in our study is in line with previous reports identifying intensive care environments as key reservoirs for resistant pathogens. Factors such as invasive procedures, indwelling medical devices, and high antibiotic pressure create a favorable setting for the emergence and persistence of opportunistic MDR organisms [37]. MDR patients had substantially longer hospital stays (median: 14 days [IQR: 9–23] vs. 6 days [IQR: 4–9], *p* < 0.0001) and greater ICU exposure (median: 0 days [IQR: 0–3] vs. 0 days [IQR: 0–0], *p* < 0.0001), reflecting increased clinical complexity and resource utilization. In the multivariate model, both ICU length of stay and total hospitalization duration remained independent predictors of MDR infection, with each additional ICU day increasing MDR odds (aOR = 1.126, 95% CI: 1.073–1.181, *p* < 0.0001), and each additional hospitalization day increasing MDR (aOR = 1.136, 95% CI: 1.113–1.160, *p* < 0.0001). These findings reinforce the well‐established relationship between prolonged healthcare exposure and nosocomial resistance, and mirror results from surgical and ICU cohorts across Europe and globally [38, 39]. Importantly, ICU stay was also demonstrated the strongest prognostic performance for in‐hospital mortality in our ROC analysis (AUC = 0.901; 95% CI: 0.867–0.934;*p* < 0.0001), supporting its value as a surrogate of disease severity and clinical deterioration. By comparison, age showed fair discrimination (AUC = 0.721; 95% CI: 0.684–0.758; *p* < 0.0001), whereas total hospitalization duration had limited predictive utility (AUC = 0.555; 95% CI: 0.499–0.611; *p* = 0.022), likely reflecting influences unrelated to fatal outcome [38].

Correlation analyses further underscored the contribution of intensive care to overall inpatient time: ICU stay was positively correlated with total hospitalization in both cohorts, with a stronger association in MDR patients (*r* = 0.2314 vs. 0.1103; both *p* < 0.0001), consistent with more protracted recovery trajectories in resistant infections.

A male predominance was observed among MDR cases (67.03% vs. 52.93%; *p* < 0.0001). Although this pattern is not consistently emphasized in the literature, it may reflect sex‐related differences in surgical case mix, baseline risk profiles, or healthcare exposure; further prospective analyses are needed to clarify whether sex represents a true biological or system‐level determinant in this setting. In contrast, place of residence was not associated with MDR status (*p* = 0.865), suggesting that MDR acquisition in this cohort was primarily driven by in‐hospital factors rather than rural/urban background.

Regarding comorbidity burden, patients with ≥ 2 comorbidities accounted for a significantly higher proportion of MDR infections in unadjusted analyses (16.76% vs. 10.42%; *p* = 0.0007), supporting the role of cumulative disease burden as a clinically relevant risk marker. Notably, in the adjusted model, ≥ 2 comorbidities were associated with lower odds of MDR infection (aOR = 0.414, 95% CI: 0.281–0.610,*p* < 0.0001). This apparent inversion suggests that comorbidity burden may capture heterogeneous care pathways rather than functioning as a direct biological determinant of MDR acquisition. Neoplasms prevalence did not differ significantly between groups (13.78% vs. 10.38%; *p* = 0.057), with benign neoplasms predominating in both cohorts.

COVID‐19 positivity was significantly more frequent in the MDR group (11.89% vs. 3.76%; *p* < 0.0001), consistent with evidence of pandemic‐associated shifts in antimicrobial exposure, immune dysregulation, and prolonged hospital trajectories [15]. However, after adjustment, COVID‐19 was not independently associated with MDR infection (aOR = 1.325, 95% CI: 0.780–2.250,*p* = 0.298), suggesting that its bivariate association may be driven by confounding through hospitalization duration, ICU exposure, and illness severity rather than a direct effect.

Perioperative antibiotic prophylaxis differed strikingly between groups: only 3.24% of MDR patients received prophylaxis compared with 54.50% of non‐MDR patients (*p* < 0.0001), while absence of prophylaxis was far more common in MDR cases (96.76% vs. 45.50%). This pronounced imbalance indicates substantial differences in perioperative antimicrobial exposure and highlights the importance of consistent stewardship oversight in surgical settings. In the adjusted model, prophylaxis was strongly associated with MDR status (aOR = 21.361, 95% CI: 11.764–38.789, *p* < 0.0001). Importantly, this finding is best interpreted as confounding by indication and case mix: perioperative antibiotics may be administered preferentially in clinically complex procedures or higher‐risk patients, who also have greater exposure to MDR acquisition through prolonged hospitalization, ICU care, or subsequent broad‐spectrum therapy, rather than implying a causal relationship. Therefore, rather than concluding that prophylaxis increases resistance, these data emphasize the need to audit timing, indication, and guideline concordance of perioperative regimens within an antimicrobial stewardship framework [39].

In parallel with these clinical predictors, our study revealed distinct patterns of antibiotic utilization between MDR and non‐MDR infections, both in frequency and duration across pharmacological classes. These patterns reflect the global challenge of diminishing antibiotic efficacy, as increasing AMR continues to limit available therapeutic options and necessitate the development of new strategies. Advances in genomic and computational approaches, including reverse vaccinology and proteomic screening, have been proposed to identify novel drug and vaccine targets by focusing on pathogen‐specific essential proteins involved in critical biological pathways [40, 41]. Such approaches facilitate the development of more effective and targeted antimicrobial therapies and highlight the importance of integrating innovative therapeutic strategies with antimicrobial stewardship efforts to address the growing burden of AMR. Aminopenicillins combined with *β*‐lactamase inhibitors constituted the most frequently prescribed class in both groups (*n* = 182 MDR vs. *n* = 675 non‐MDR), with longer treatment durations in MDR cases (median: 5.5 [3–7] vs. 4 [3–6] days), reflecting more complex courses and potential delays in effective source control. Nitroimidazoles and third‐generation cephalosporins were used more frequently in non‐MDR infections but were administered for longer durations in MDR cases, consistent with more severe or prolonged intra‐abdominal and polymicrobial infections. In contrast, prolonged use of carbapenems, glycopeptides, oxazolidinones, glycylcyclines, and polymyxins in MDR patients underscores the dependence on broader‐spectrum and last‐resort agents when therapeutic options are constrained. These patterns were echoed in perioperative and intraoperative antibiotic use: second‐generation cephalosporins dominated perioperative prophylaxis among non‐MDR patients (*n* = 665 vs. *n* = 10 in MDR), while intraoperative administration in MDR cases favored aminopenicillin + *β*‐lactamase inhibitors and second‐generation cephalosporins. Overall, this prescribing profile suggests a dual influence of resistance ecology and stewardship constraints on real‐world antimicrobial selection in surgical care.

From a microbiological perspective, the pathogen spectrum was broad and heterogeneous. Overall, *E. coli* was the most common isolated (*n* = 209), consistent with its central role in postoperative urinary and intra‐abdominal infections. *K. pneumoniae* ranked second (*n* = 126), reflecting its established role as a nosocomial pathogen and reservoir for ESBL and carbapenemase‐mediated resistance. Nonfermenters such as *P. aeruginosa* (*n* = 67) and *A. baumannii* (*n* = 30) were also prominent, particularly relevant for healthcare‐ and ICU‐associated infections given their intrinsic resistance and propensity for acquiring additional determinants. Among Gram‐positive organisms, *C. difficile* was frequently detected (*n* = 59), consistent with substantial antibiotic exposure and intestinal microbiota disruption in surgical populations. *S. aureus* (*n* = 52), including MRSA, and enterococci, especially *E. faecalis* (*n* = 46) and *E. faecium* (*n* = 37), remained clinically important, reinforcing their roles in SSI, BSI, and device‐associated infections. Notably, more than 50 additional species were recovered at lower frequencies, including *R. planticola*, *Actinomyces* spp., and *B. fragilis*, highlighting the polymicrobial complexity of surgical infections and supporting the continued need for tailored antibiograms and ongoing surveillance [39, 42, 43].

Antibiotic susceptibility profiles showed preserved activity of aminoglycosides, polymyxins, and carbapenems against Gram‐negative MDR organisms, while glycopeptides and oxazolidinones retained activity against MDR Gram‐positive isolates. Conversely, older or narrow‐spectrum classes, including penicillins and tetracyclines, demonstrated limited utility, consistent with contemporary global resistance profiles [29, 34, 44]. At the same time, the substantial proportion of multisusceptible isolated (*n* = 448) suggests that a meaningful subset of infections may represent earlier‐onset or less antibiotic‐exposed trajectories, emphasizing the clinical value of timely diagnostics, targeted therapy and stewardship‐aligned prescribing [39, 44]. Although MDR prevalence in this cohort was lower than that reported in some high‐burden African settings (e.g., > 75% in Ethiopian SSI) [43], it exceeded Romania′s national average (~20% in HAIs) [39], underscoring the need for strengthened local stewardship and infection prevention measures.

In summary, our findings indicate that healthcare exposure, particularly ICU stay and prolonged hospitalization, is the most robust independent predictor of MDR infection in surgical patients. While age, COVID‐19 positivity, comorbidity burden, and neoplastic disease showed associations in unadjusted analyses, these relationships changed after multivariate adjustment, highlighting the dominant role of inpatient exposure and clinical complexity. The multivariate model demonstrated good overall fit (Nagelkerke *R* ^2^ = 0.463) and high specificity (96.7%) but limited sensitivity (35.4%), indicating that it performs better for ruling out MDR risk than for identifying all MDR cases. Additional predictors, potentially including procedure complexity, device exposure, prior antibiotic history, and timing of cultures, may improve discrimination. Strengthening perioperative antimicrobial practice, optimizing stewardship oversight, and investing in ward‐specific surveillance systems remain essential to mitigate MDR burden and improve outcomes in tertiary surgical care.

Our findings are broadly consistent with reports from other European surgical and ICU cohorts, reinforcing their relevance within the continental AMR landscape [5, 45]. Studies from Southern and Central Europe, including Italy [46], Greece [47, 48], and Poland [49, 50], have similarly identified prolonged hospitalization/ICU exposure and extensive antimicrobial use as principal drivers of MDR acquisition, with *K. pneumoniae*, *P. aeruginosa*, and *A. baumannii* commonly predominating among resistant isolates.

Comparable European analyses also document a disproportionate reliance on reserve/broad‐spectrum agents in MDR contexts, while more conventional regimens remain common in non‐MDR or prophylactic settings [5, 45]. The MDR prevalence observed in our cohort, exceeding some national benchmarks yet remaining below rates reported in certain Southern European ICU‐focused studies, likely reflects differences in case mix, surgical complexity, baseline ICU burden, and local stewardship implementation [5, 46, 48].

Collectively, these parallels indicate that the resistance patterns and prescribing behaviors observed in our Romanian tertiary‐care surgical unit align with broader European trends, underscoring shared challenges and the need for coordinated stewardship strategies across surgical settings [5, 45].

## 5. Conclusions

This study provides a comprehensive assessment of the clinical, microbiological, and therapeutic determinants of MDR infections among surgical inpatients in a Romanian tertiary‐care hospital. MDR infections were independently associated with a markedly increased risk of death, even after adjustment for age, ICU stay, and hospitalization duration, underscoring the clinical significance of AMR beyond disease severity alone. Among all predictors, ICU exposure emerged as the strongest determinant of both MDR acquisition and mortality, highlighting the central role of critical illness and healthcare‐associated factors. Prolonged hospitalization and ICU stay remained the most robust predictors of MDR acquisition, confirming the importance of cumulative healthcare exposure in resistance development. While age, comorbidity burden, and prior SARS‐CoV‐2 infection were associated with MDR infection in univariate analyses, their effects were attenuated after multivariate adjustment, suggesting that they are mediated through disease severity and healthcare utilization.

Marked differences in antibiotic utilization patterns were observed between MDR and non‐MDR patients. MDR infections required prolonged exposure to broad‐spectrum and last‐line agents, including carbapenems, glycopeptides, oxazolidinones, and polymyxins, whereas non‐MDR patients were predominantly managed with second‐generation cephalosporins and aminopenicillin/*β*‐lactamase inhibitor combinations. These findings reflect both the increased clinical complexity of MDR infections and constrained therapeutic options available once resistance is established.

Importantly, the association between perioperative antibiotic prophylaxis and MDR status likely reflects confounding by indication rather than a direct causal effect, emphasizing the need for careful stewardship evaluation of timing, indication, and guideline adherence.

Overall, these results highlight the need for strengthened antimicrobial stewardship strategies, particularly in ICU and surgical settings, early identification of high‐risk patients, and optimization of antibiotic exposure. Integrating clinical risk prediction models, stewardship oversight, and local epidemiological surveillance may contribute to reducing the burden of MDR infections and improving patient outcomes.

## 6. Strengths of the Study

This study has several notable strengths that enhance its scientific relevance and clinical applicability. First, it represents one of the relatively few investigations from Eastern Europe specifically addressing MDR infections in a surgical inpatient population, a setting that remains underrepresented in global AMR literature. By integrating detailed clinical characteristics, microbiological profiles, and antibiotic utilization patterns, the analyses provide a multidimensional assessment of MDR acquisition and its therapeutic consequences. A major strength lies in the granular evaluation of antimicrobial use across resistance profiles, including perioperative and intraoperative administration, with class‐specific analyses of frequency and duration. This approach allows differentiation between prophylactic and therapeutic exposure and offers real‐world insight into stewardship practices within surgical care pathways. Methodologically, the use of multivariate logistic regression enabled the identification of independent predictors of both MDR infection and in‐hospital mortality, while ROC curve analysis strengthened the prognostic evaluation of clinical variables, particularly ICU stay as a marker of disease severity.

Taken together, our findings align with the broader global understanding of AMR as a multifactorial phenomenon driven by healthcare exposure, pathogen adaptability, and antibiotic selection pressure. The convergence of clinical, microbiological, and therapeutic factors observed in our cohort underscores the urgent need for integrated stewardship strategies, continuous surveillance, and the development of novel antimicrobial approaches to mitigate the impact of MDR infections.

## 7. Limitations of the Study

Despite these strengths, several limitations should be acknowledged. The retrospective, single‐center design inherently limits external generalizability, as antibiotic prescribing patterns, microbiological epidemiology, and stewardship policies may differ across institutions and healthcare systems. Microbiological data were derived from routine clinical diagnostics, which may underestimate colonization or infection with slow‐growing, fastidious, or uncultured MDR organisms. Additionally, detailed information regarding the appropriateness, timing, and guideline adherence of antibiotic prescriptions, particularly perioperative prophylaxis, was not consistently available, precluding a formal evaluation of compliance with stewardship guidelines. Residual confounding cannot be excluded, as data on prior outpatient antibiotic exposure, baseline colonization status at admission, and standardized comorbidity indices were not systematically recorded. Moreover, while comorbidity burden was incorporated as a binary variable to reduce multicollinearity, this approach may oversimplify the heterogeneous biological impact of individual chronic conditions.

Nevertheless, within these constraints, the study provides clinically meaningful and actionable insights. The findings support targeted antimicrobial stewardship interventions, optimization of surgical prophylaxis strategies, and early identification of high‐risk patients for MDR acquisition. Future multicentric, prospective studies incorporating standardized stewardship metrics, antimicrobial pharmacodynamics, molecular resistance profiling, and patient‐centered outcomes are warranted to validate and expand these results and to inform evidence‐based policies in surgical settings.

**Nomenclature**
MDR: multidrug‐resistant
ROC: receiver operating characteristic
ICU: intensive care unit
AMR: antimicrobial resistance
GLASS: Antimicrobial Resistance and Use Surveillance System
BSI: bloodstream infection
UTI: urinary tract infection
EU/EEA: European Union/European Economic Area
ECDC: European Centre for Disease Prevention and Control
WGS: whole‐genome sequencing
LMICs: low‐ and middle‐income countries
HAI: hospital‐acquired infection
SSI: surgical site infection
WHO: World Health Organization
ESBLs: extended‐spectrum *β*‐lactamases
MRSA: methicillin‐resistant *Staphylococcus aureus*
ESKAPE: *Enterococcus faecium*, *Staphylococcus aureus*, *Klebsiella pneumoniae*, *Acinetobacter baumannii*, *Pseudomonas aeruginosa*, and *Enterobacter* spp. (a group of highly resistant pathogens)
EUCAST: European Committee on Antimicrobial Susceptibility Testing
AST: antimicrobial susceptibility testing
MALDI‐TOF MS: matrix‐assisted laser desorption/ionization time‐of‐flight mass spectrometry
IQR: interquartile range
VIF: variance inflation factor
AIC: Akaike information criterion
BIC: Bayesian information criterion
aOR: adjusted odds ratio
CI: confidence interval
AUC: area under the curve
OR: odds ratio
BCa: bias‐corrected and accelerated (bootstrap confidence interval method)

## Author Contributions

Conceptualization and methodology: L.A., M.D., O‐D.I., L.I., and D‐V.T. Formal analysis, investigation, data curation, visualization: O‐D.I. Writing—original draft preparation and writing—review and editing: L.A., M.D., O‐D.I., T‐D.C., M.P., M.B., L.I., and D‐V.T. All authors contributed to the article.

## Funding

No funding was received for this manuscript.

## Disclosure

All authors approved the submitted version.

## Ethics Statement

This study was approved by the Ethics Committee of the “Grigore T. Popa” University of Medicine and Pharmacy, Iasi (approval no. 597/3.05.2025).

## Consent

The authors have nothing to report.

## Conflicts of Interest

The authors declare no conflicts of interest.

## Supporting information


**Supporting Information** Additional supporting information can be found online in the Supporting Information section. Figure S1: Distribution of (A) ICU length of stay and (B) total hospitalization duration in patients with MDR and non‐MDR infections. Data are presented as box‐and‐whisker plots, with central lines indicating the median, boxes representing the IQR, and whiskers extending from the 10th to the 90th percentiles; individual points correspond to single patients. Group comparisons were performed using the Mann–Whitney *U* test due to non‐normal distribution of data; ∗∗∗∗*p* < 0.0001. MDR classified individuals experienced markedly prolonged ICU stays and total hospitalization durations compared with non‐MDR patients. These differences were highly significant and reflected a broader dispersion and higher upper range of values among MDR cases, consistent with increased clinical complexity and resource utilization.

## Data Availability

The data that support the findings of this study are available from the corresponding author upon reasonable request.
