# Supplementary figures and images for: Risk Factors and Antibiotic Utilization Patterns in Multidrug‐Resistant Surgical Infections: A Retrospective Study From a Romanian Tertiary‐Care Center
Source: Int J Microbiol. 2026 Jun 19;2026:6286424. doi: 10.1155/ijm/6286424 (PMC13282456; doi:10.1155/ijm/6286424)

**A**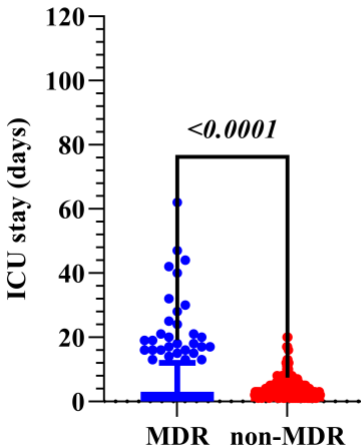

Supplement: Supplementary file 1 — Supporting Information Additional supporting information can be found online in the Supporting Information section. Figure S1: Distribution of (A) ICU length of stay and (B) total hospitalization duration in patients with MDR and non‐MDR infections. Data are presented as box‐and‐whisker plots, with central lines indicating the median, boxes representing the IQR, and whiskers extending from the 10th to the 90th percentiles; individual points correspond to single patients. Group comparisons were performed using the Mann–Whitney U test due to non‐normal distribution of data; ∗∗∗∗p < 0.0001. MDR classified individuals experienced markedly prolonged ICU stays and total hospitalization durations compared with non‐MDR patients. These differences were highly significant and reflected a broader dispersion and higher upper range of values among MDR cases, consistent with increased clinical complexity and resource utilization. [file IJM-2026-6286424-s001.zip › Supplementary Figure S1 A.pdf]

# B

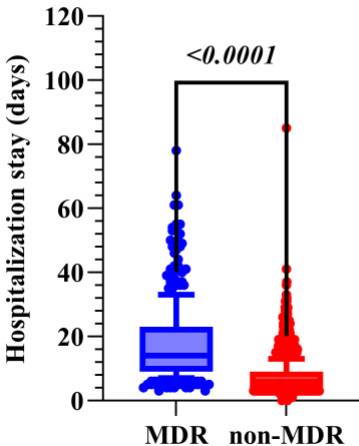

Supplement: Supplementary file 1 — Supporting Information Additional supporting information can be found online in the Supporting Information section. Figure S1: Distribution of (A) ICU length of stay and (B) total hospitalization duration in patients with MDR and non‐MDR infections. Data are presented as box‐and‐whisker plots, with central lines indicating the median, boxes representing the IQR, and whiskers extending from the 10th to the 90th percentiles; individual points correspond to single patients. Group comparisons were performed using the Mann–Whitney U test due to non‐normal distribution of data; ∗∗∗∗p < 0.0001. MDR classified individuals experienced markedly prolonged ICU stays and total hospitalization durations compared with non‐MDR patients. These differences were highly significant and reflected a broader dispersion and higher upper range of values among MDR cases, consistent with increased clinical complexity and resource utilization. [file IJM-2026-6286424-s001.zip › Supplementary Figure S1 B.pdf]
